# Challenges and prospects of plasmonic metasurfaces for photothermal catalysis

**DOI:** 10.1515/nanoph-2022-0073

**Published:** 2022-05-23

**Authors:** Luca Mascaretti, Andrea Schirato, Paolo Fornasiero, Alexandra Boltasseva, Vladimir M. Shalaev, Alessandro Alabastri, Alberto Naldoni

**Affiliations:** Czech Advanced Technology and Research Institute, Regional Centre of Advanced Technologies and Materials, Palacký University Olomouc, Šlechtitelů 27, 77900 Olomouc, Czech Republic; Department of Physics, Politecnico Di Milano, Piazza Leonardo Da Vinci 32, 20133 Milan, Italy; and Istituto Italiano di Tecnologia, Via Morego 30, 16163 Genoa, Italy; Department of Chemical and Pharmaceutical Sciences, Center for Energy, Environment and Transport Giacomo Ciamiciam, INSTM Trieste Research Unit and ICCOM-CNR Trieste Research Unit, University of Trieste, Via L. Giorgieri 1, 34127 Trieste, Italy; School of Electrical & Computer Engineering and Birck Nanotechnology Center, Purdue University, West Lafayette, USA; Department of Electrical and Computer Engineering, Rice University, 6100 Main Street, 77005 Houston, TX, USA

**Keywords:** gas phase, photocatalysis, photothermal catalysis, plasmonic metasurfaces, solar fuels

## Abstract

Solar-thermal technologies for converting chemicals using thermochemistry require extreme light concentration. Exploiting plasmonic nanostructures can dramatically increase the reaction rates by providing more efficient solar-to-heat conversion by broadband light absorption. Moreover, hot-carrier and local field enhancement effects can alter the reaction pathways. Such discoveries have boosted the field of photothermal catalysis, which aims at driving industrially-relevant chemical reactions using solar illumination rather than conventional heat sources. Nevertheless, only large arrays of plasmonic nano-units on a substrate, i.e., plasmonic metasurfaces, allow a quasi-unitary and broadband solar light absorption within a limited thickness (hundreds of nanometers) for practical applications. Through moderate light concentration (∼10 Suns), metasurfaces reach the same temperatures as conventional thermochemical reactors, or plasmonic nanoparticle bed reactors reach under ∼100 Suns. Plasmonic metasurfaces, however, have been mostly neglected so far for applications in the field of photothermal catalysis. In this Perspective, we discuss the potentialities of plasmonic metasurfaces in this emerging area of research. We present numerical simulations and experimental case studies illustrating how broadband absorption can be achieved within a limited thickness of these nanostructured materials. The approach highlights the synergy among different enhancement effects related to the ordered array of plasmonic units and the efficient heat transfer promoting faster dynamics than thicker structures (such as powdered catalysts). We foresee that plasmonic metasurfaces can play an important role in developing modular-like structures for the conversion of chemical feedstock into fuels without requiring extreme light concentrations. Customized metasurface-based systems could lead to small-scale and low-cost decentralized reactors instead of large-scale, infrastructure-intensive power plants.

## 1 Introduction

The so-called “Glasgow Climate Pact” resulting from the recent 2021 United Nations Climate Change Conference (COP26) reaffirmed the crucial importance of limiting global warming below 2 °C above pre-industrial levels, possibly below 1.5 °C as this would imply much lower impacts of climate change [1]. However, such action requires exceptional efforts to reduce greenhouse gas emissions: by 2050 the World should reach net carbon neutrality. Such aims demand rapid innovations in the technologies employed to generate energy, fuels, and chemicals.

**Figure 1:**
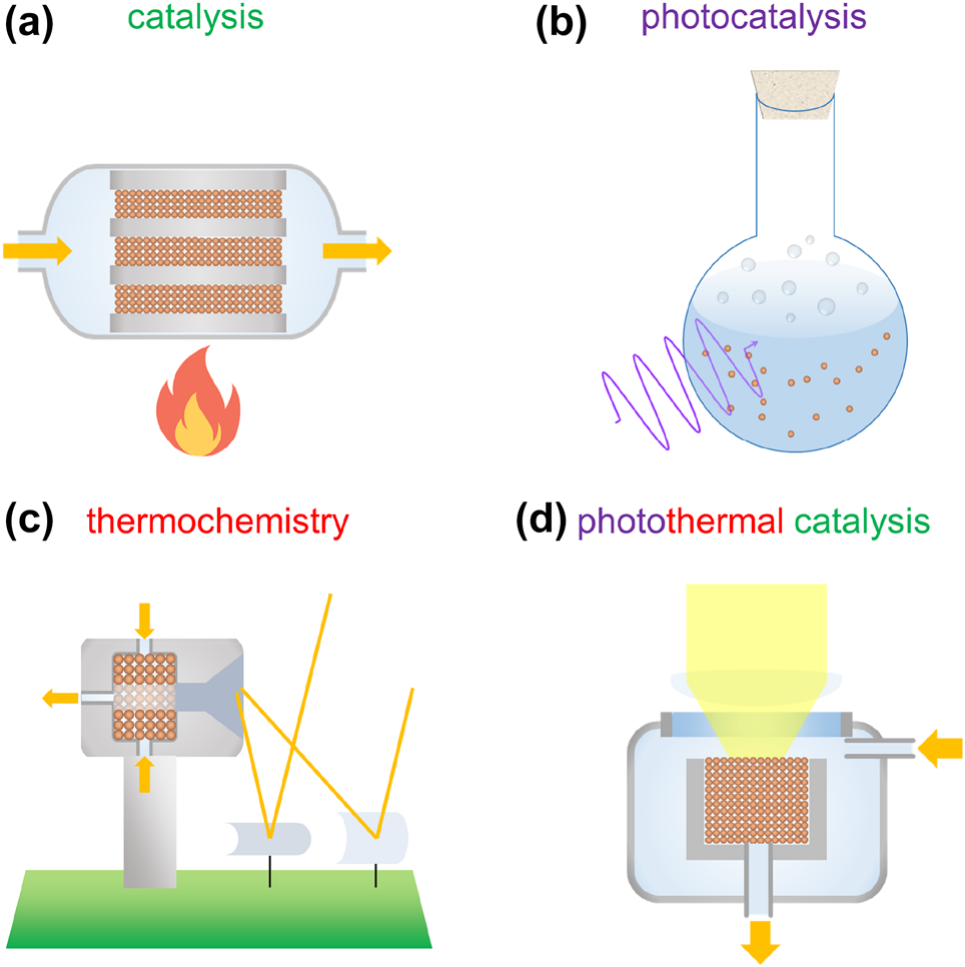
Schematic illustration of (a) catalysis, (b) photocatalysis, and (c) thermochemistry, which, combined together, lead to the approach of (d) photothermal catalysis.

One century ago, a technological breakthrough allowed humans to evade a worrying situation: the depletion of natural supplies of nitrogen-based fertilizers was threatening humanity with starvation, but the discovery of the nitrogen fixation process to ammonia enabled large-scale production of such fertilizers. This process is known as Haber–Bosch process (N_2_ + 3H_2_ → 2NH_3_, Δ*G*_f_^°^ = −16.4 kJ mol^−1^) and it represents one of the most important examples of heterogeneous catalysis [2]. Industrial reactors for heterogeneous catalysis (Figure 1a) consist of containers operating at high temperatures (>400 °C) and pressures (>100 atm) accommodating a catalyst bed that is exposed to the reactant gases, to be transformed into the desired products. The presence of a suitable catalyst (such as Fe-based ones for the ammonia synthesis) and suitable temperature/pressure conditions in the reactor allows the specific chemical reaction to proceed optimally. This process would not occur under environmental conditions despite being thermodynamically spontaneous. Catalysis plays an irreplaceable role in modern society because it allows producing, besides fertilizers, fuels, pharmaceuticals, industrial chemicals and plastics. If the energy necessary to drive the catalytic reactions could be provided by renewable sources, a significant step toward carbon neutrality would be made.

In this regard, the Sun provides us with 120,000 TW of continuous radiation and, in principle, represents an almost unlimited renewable energy source compared to today’s global average power consumption of 19 TW [3, 4]. The idea of using sunlight to drive catalytic processes is not new: the so-called photocatalysis was proposed already in the early years of the 20th century [5]. This field then dramatically grew in the 1970s, spanning many applications, including the generation of fuels (such as hydrogen from water), chemicals, and the degradation of organic pollutants. However, typical photocatalysis experiments (Figure 1b) differ from industrial catalysis because they involve a semiconductor in powdered form (such as TiO_2_, Cu_2_O, Fe_2_O_3_, WO_3_, C_3_N_4_) dispersed in an aqueous solution at ambient temperature containing the molecules to be transformed [6]. Semiconductor photocatalysts absorb the light energy and transform it into charge carriers to drive both thermodynamically spontaneous and non-spontaneous reactions (as in natural photosynthesis). An important example is the very well-known case of photocatalytic water splitting (H_2_O → H_2_ + 1/2O_2_, Δ*G*_f_^°^ = 237 kJ mol^−1^). However, despite decades of research, only a few photocatalysis applications have been developed to the industrial scale, such as self-cleaning coatings on windows or photoactive cements [7], essentially due to material limitations.

An alternative strategy that exploits sunlight as a source of energy to produce fuels and chemicals with great scale-up potential is thermochemistry. The thermochemical approach takes advantage of the already developed reactor design of the concentrated solar power (CSP) technology, which employs mirrors or lenses to concentrate light up to regimes >2000 times on a collector to reach temperatures exceeding 1000 °C [8]. In the case of thermochemistry (Figure 1c), the collector contains a reducible oxide (such as ceria, CeO_2_) which, at such high temperatures, undergoes an endothermic reduction to a lower valence state, releasing oxygen (CeO_2_ → CeO_2–δ_ + δ/2O_2_). The reduced oxide then reacts endothermically with H_2_O to form H_2_ (CeO_2−δ_ + δH_2_O → CeO_2_ + δH_2_) or with CO_2_ to form CO (CeO_2−δ_ + δCO_2_ → CeO_2_ + δCO), thus returning to the initial oxidation state [9, 10]. Overall, such two-step thermochemical cycle allows converting H_2_O and CO_2_ extracted from air into syngas (i.e., mixtures of CO and H_2_ with various ratios) as a suitable product for further transformation into methanol or other liquid hydrocarbons as fuels [11].

The key features discussed above for catalysis, photocatalysis, and thermochemistry can be incorporated in a unified approach that can be defined as photothermal catalysis (Figure 1d) [12, 13]. This strategy aims at efficiently driving either exothermic (i.e., ammonia synthesis and CO_2_ reduction) or endothermic (i.e., water splitting) reactions by combining the photocatalytic and solar-thermal mechanisms without the need of extreme concentration regimes to reach high temperatures. This can be achieved by combining more materials into fully-metallic or composite oxide/metallic photocatalysts characterized by a high absorption across the whole solar spectrum, efficient solar-thermal conversion and high catalytic activity. One of the first reported examples consisted in the use of Ru nanoparticles (NPs) supported by silicon nanowires (Ru@SiNW) for the Sabatier methanation reaction (CO_2_ + 4H_2_ → CH_4_ + 2H_2_O), using light intensity in the range of 10–20 Suns (1 Sun = 1 kW m^−2^) [14]. Since photothermal reactions with semiconductor photocatalysts usually produce a limited increase in temperature, and such materials suffer a bandgap reduction with increasing temperature [15], scientists have increasingly considered metal nanostructures supporting surface plasmon resonances, leading to the development of the sub-field of plasmonic photothermal catalysis [16, 17]. A crucial advantage in this regard is the possibility of exploiting plasmonic effects to access excited states of the reactant-catalyst system associated with a different activation energy or even different products compared to the same chemical process at the ground state.

Most of the studies on photothermal catalysis reported so far considered catalysts in powdered forms, which require a few mm to reach complete solar light absorption and light intensities >10 Suns to generate appreciable reaction rates, even in the presence of plasmonic nanostructures. Further developments are thus expected to be achieved by rather considering plasmonic metasurfaces, i.e., periodically ordered two-dimensional arrays of sub-wavelength units [18, 19]. These systems support complex electromagnetic resonances that facilitate the achievement of broadband light absorption in a sub-µm thickness and that often lead to unique nonlinear optical phenomena resulting in enhanced hot carrier generation and substantial heating promoted by collective photothermal effects under moderate light concentration (∼10 Suns) [20], [21], [22]. While traditional applications of metasurfaces have been almost restricted to nanophotonics [19, 23, 24], such nanopatterned materials have been increasingly considered for solar-energy harvesting, such as in thermophotovoltaics [25] and steam generation [26, 27]. Few recent studies have convincingly shown that metasurfaces are also valuable candidates in photoelectrochemistry and gas-phase photothermal catalysis [28], [29], [30].

This Perspective is organized as follows Section 2 reports a brief overview on photothermal catalysis with powdered plasmonic systems, highlighting the thermal and non-thermal plasmonic mechanisms and timescales (Section 2.1) and discussing how such mechanisms have been selectively or synergistically employed to enhance the reaction rate and selectivity (Section 2.2). Section 3 details the general properties of plasmonic metasurfaces, their potential advantages compared to conventional powdered plasmonic systems, and presents numerical simulations to show how the optical broadband absorption could be engineered. Section 4 illustrates early examples and more recent improvements of plasmonic metasurfaces for photothermal catalysis and photoelectrochemistry. Section 5 finally summarizes the potentialities of metasurfaces as well as critical factors that should be improved in view of realistic implementation in the field of photothermal catalysis, including the fabrication process, materials selection, time-resolved and theoretical studies, appropriate benchmarking, and reactor design.

## 2 Photothermal catalysis with powdered plasmonic systems

Most of the studies on photothermal catalysis have considered so far NP-based porous pellets that closely resemble the catalysts employed in the industry. In this regard, several studies focused on photothermal CO_2_ reduction mechanisms driven by transition metal catalytic NPs supported on oxides [31], [32], [33], [34], [35]. This topic has been recently discussed in a dedicated review [36]. In this Section, we instead focus on the enhancement mechanisms produced by plasmonic nanostructures illustrating the potential of such effects in tuning the product selectivity of various chemical reactions.

### 2.1 Fundamental mechanisms and timescales

**Figure 2:**
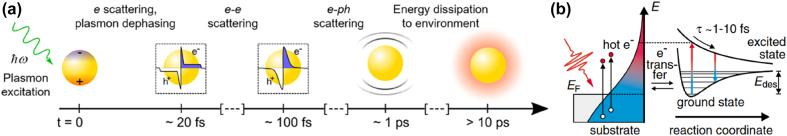
Mechanisms and timescales of plasmon decay. (a) Characteristic timescales and phenomena related to plasmon excitation and decay. Adapted with permission from ref. [39]. Copyright 2019, Elsevier. (b) Schematic of the electron transfer process from a plasmonic NP to the unoccupied state of an adsorbed molecule. Adapted with permission from ref. [42]. Copyright 2003, American Physical Society.

Surface plasmon resonances consist of collective electron oscillations coupled with the incident light and can be classified into (i) surface plasmon polaritons (SPPs), i.e., bi-evanescent electron oscillations propagating along the interface between a metal and a dielectric; and (ii) localized surface plasmons (LSPs), i.e., bounded driven electron oscillations in confined nanostructures [37]. These resonances induce a plethora of effects at different time scales (Figure 2a) which, either individually or synergistically, can drive or enhance chemical reactions. Plasmon excitation follows light absorption and results into strong local electric field enhancements at the nanostructure surface, especially in the presence of sharp edges (i.e., in nanocubes or nanostars). The plasmon dephasing takes place in tens of femtoseconds either radiatively (i.e., scattering effect, favored in nanostructures larger than 50 nm) or non-radiatively (favored in smaller nanostructures), thus generating energetic (hot) carriers with excess energy up to the photon energy. In turn, these out-of-equilibrium carriers undergo internal non-linear relaxation processes over well-distinct timescales toward equilibrium [38], [39], [40]. In the first hundreds of fs, the absorbed energy is redistributed *via* scattering events within the metal electronic population. Electrons (holes) are promoted to excited energetic states above (below) the Fermi level, from which they may be injected to unoccupied (occupied) orbitals of an adsorbed reactive molecule. This charge injection effect, usually referred to as hot electron (hole) transfer, is likely to be the most interesting mechanism in plasmon-based photothermal catalysis, because it can move the reactant-catalyst system to a different potential energy surface (PES) compared to the ground state one (Figure 2b). Alternative, non-thermal (since mediated by hot carriers), reaction pathways become available, along which the excited system could experience a different activation energy or a different minimum for the products compared to those at the ground state. This mechanism competes with the typical timescale for activating and breaking a chemical bond inside a molecule, i.e., a few picoseconds, as first demonstrated by pump-probe experiments on metallic single crystals under intense laser excitation [41, 42]. If no charge transfer occurs, excited carriers equilibrate with the metal lattice *via* electron-phonon scattering within a few ps. This results in an increased temperature of the metal. Finally, heat is released *via* phonon-phonon scattering towards the environment on a timescale from a few nanoseconds until the stationary regime, which leads to heating effects in the environment surrounding the nano-object. Such a light-induced local temperature increase can drive the catalytic reaction along the ground state PES, similarly to conventional (dark) heterogeneous catalysis. Since chemical reactions occur in a wide range of timescales, i.e., from µs to several s, heating can provide a beneficial effect irrespectively of the specific catalyst/reactant system.

Our current understanding of the ultrafast energy relaxation processes following plasmon dephasing as detailed above has been achieved by a combination of experimental and theoretical investigations of the photothermal response of plasmonic systems. Specifically, transient absorption spectroscopy (TAS) [43], [44], [45], [46] is among the most suitable tool, enabling the study of the energy flow governed by ultrafast hot electron dynamics. In parallel, theoretical models including fully-quantum and ab initio calculations, or thermodynamic rate-equation models have been proposed to examine the hot carrier relaxation processes and injection, providing insight into the fundamental mechanisms presiding over the photoexcitation [47], [48], [49], [50]. It should also be noted that, during steady-state illumination, the processes described above occur simultaneously and could, therefore, act synergistically to enhance the rate of chemical reactions.

### 2.2 Plasmonic effects in photothermal catalysis

Photothermal catalysis experiments with powdered plasmonic systems usually evaluate the effect of plasmonic NPs in driving gas-phase industrially-relevant chemical reactions under solar light illumination or in tuning the product selectivity to avoid undesired products. Examples of such reactions are the epoxidation of ethylene [51, 52] or propylene [53], the selective hydrogenation of acetylene [54], or ammonia synthesis [55, 56]. A critical factor consists in the accurate distinction and quantification of thermal and non-thermal effects (as described in Section 2.1), which was overlooked in the first studies in this field, but has been recently acknowledged and fervently discussed [57], [58], [59], [60], [61], [62], [63], [64], [65], [66]. Guidelines in this regard have also been proposed [67], [68], [69], even though rigorous standards have not appeared yet (unlike the well-established case of the photovoltaic community).

**Figure 3:**
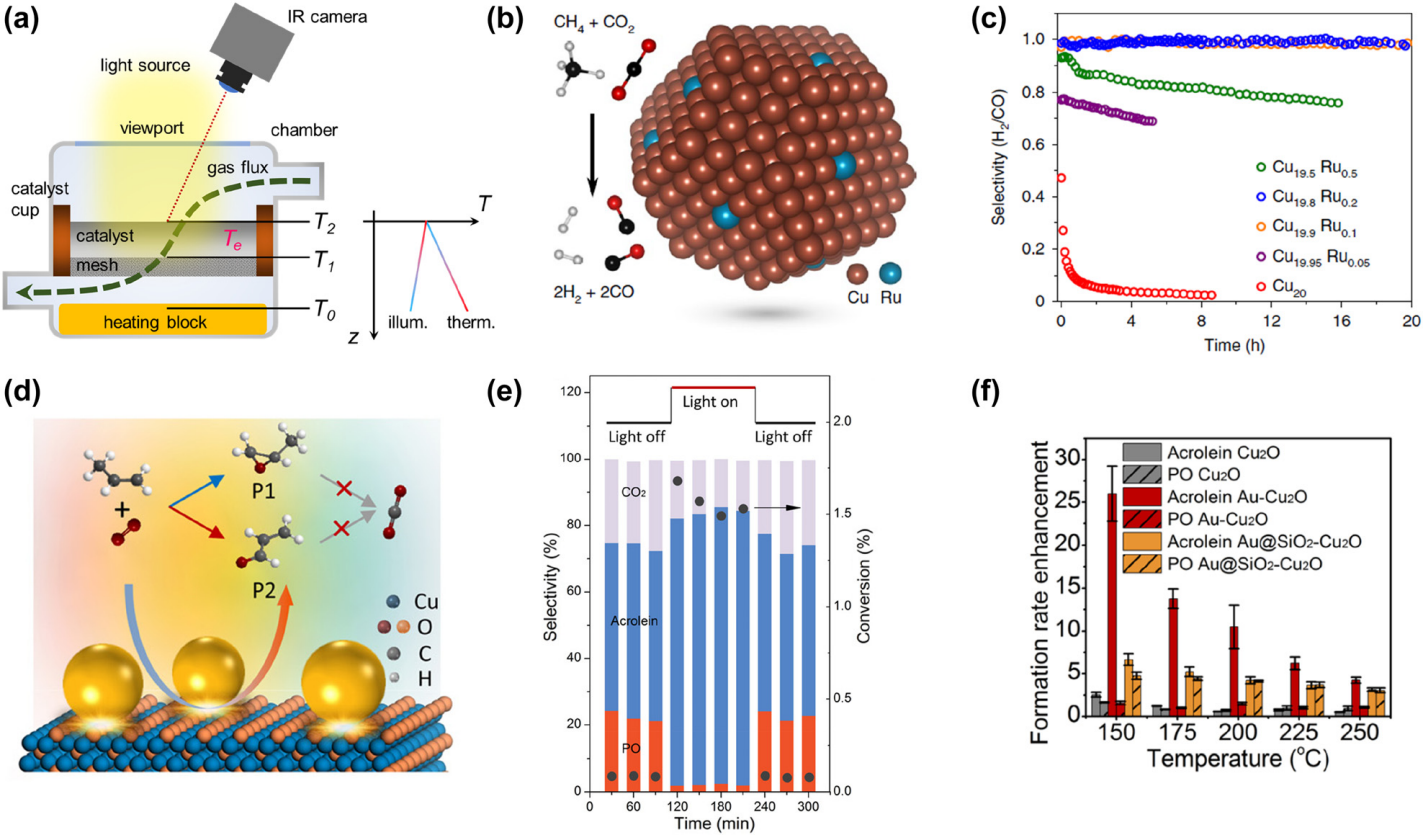
Photothermal catalysis with powdered plasmonic systems. (a) Schematic illustration of a typical reactor setup for plasmonic photocatalysis experiments, employing different thermocouples (*T*_0_, *T*_1_, and *T*_2_) as well as non-contact techniques to detect thermal gradients in the catalyst bed, which could differ depending on the heating source (either the light irradiation or the heater block). Adapted with permission from ref. [67]. Copyright 2020, AIP Publishing. (b) Schematic of Cu–Ru catalyst for the methane dry reforming reaction and (c) selectivity toward the different products under 19.2 W cm^−2^ as a function of the Ru concentration. Adapted with permission from ref. [70]. Copyright 2020, Springer Nature. (d) Schematic of the partial oxidation of propylene to acrolein (P1) and propylene oxide (P2) on Au–Cu_2_O plasmonic structure, (e) selectivity and conversion to those products at 150 °C with and without illumination, and (f) effect of coating the Au NPs with a 5 nm-thick SiO_2_ shell on the formation rate of the products. Adapted with permission from ref. [73]. Copyright 2021 Author(s), licensed under a CC-BY Creative Commons Attribution 4.0 License.

In typical plasmonic catalytic conditions, the experimental reactor is designed as a vacuum-cell enclosing the catalyst cup equipped with a main viewport, allowing catalyst irradiation (by a solar simulator, a LED, or a laser), and additional viewports for non-contact temperature measurements (through an IR sensor or thermal camera) or *in-situ* spectroscopy analysis [55] (Figure 3a). Plasmonic and/or catalytic NPs are dispersed in a metal oxide matrix in powdered form and the material is pressed into a mm-thick porous pellet to achieve catalytic activity, high surface area, and high optical absorption. The reaction rate r is usually calculated from the areas of the reaction product peaks in gas chromatograph (GC) or mass spectrometer (MS) data and is typically quantified in mmol g_cat_^−1^ h^−1^, where the subscript “cat” refers to the amount of the catalytically active phase retrieved from inductively coupled mass (ICP) spectroscopy. The activation energy for the overall photothermal process *E*_a_ (in kJ mol^−1^) can thus be derived from the reaction rate by Arrhenius equation, *r* = *r*_0_ exp(–*E*_a_/(RT)), where *r*_0_ is a constant for the given reaction, *R* is the ideal gas constant and *T* is the temperature. More products are usually formed because of the simultaneous occurrence of competitive mechanisms or side-reactions, so each product is associated with a rate *r*_1_, *r*_2_, etc. The primary goals of photothermal catalysis are to selectively yield one product of interest from the overall mechanism and/or to decrease the value of the associated *E*_a_ compared to the purely thermal process by means of non-thermal effects, such as hot electron transfer. If two main products are detected for a certain reaction mechanism, the product selectivity can be evaluated as

(1) $$S=r_{1}/r_{2}$$

or, alternatively,

(2) $$S\left(\%\right)=\left[r_{1}/\left(r_{1}+r_{2}\right)\right]\cdot 100$$

On the other hand, proving a decrease of *E*_a_ by non-thermal effects is very challenging even in the case of precise temperature measurements with thermocouples (to track thermal gradients along the catalyst thickness) and non-contact techniques (Figure 3a), as thoroughly discussed elsewhere [61], [62], [63].

In a recent example, Cu–Ru NPs were employed as antenna–reactor plasmonic photocatalysts to control the product selectivity of the methane dry reforming (MDR) reaction, CH_4_ + CO_2_ → 2CO + 2H_2_, that allows converting greenhouse gases (CH_4_ and CO_2_) into syngas (CO and H_2_ mixture) [70]. Apart from coking (i.e., graphitic carbon deposition during the reaction), the main side reaction was the reverse water gas shift (RWGS) process, CO_2_ + H_2_ → CO + H_2_O, so that the selectivity of the overall mechanism could be expressed as *S* = *r*_H2_/*r*_CO_. The photocatalyst consisted of Cu NPs of ∼5 nm diameter loaded on a MgO–Al_2_O_3_ support and modified with highly dispersed Ru atoms (Figure 3b). The maximum reaction rate of 1260 mmol g^−1^ h^−1^ and energy efficiency of ∼15% were achieved under white light illumination at 19.2 W cm^−2^ (i.e., ∼200 Suns), generating temperatures as high as ∼730 °C. In such conditions, according to DFT simulations, the LSP resonance of Cu NPs acted as antenna generating hot carriers and heating, which in turn enhanced the rate of C–H activation on the atomically-isolated Ru sites and H_2_ desorption from the NP surface. This allowed increasing the selectivity from ∼0 for pure Cu NPs to ∼1 for optimized compositions (Figure 3c), as well as suppressing the formation of graphitic compounds, leading to high stability (50 h). A similar strategy was also reported for Cu–Zn NPs supported on SiO_2_ to drive the methanol steam reforming (MSR) reaction, CH_3_OH + H_2_O → 3H_2_ + CO_2_ [71]. Under 7.9 Suns irradiation, corresponding to ∼250 °C, the maximum *r*_H2_ = 328 mmol g_cat_^−1^ h^−1^ and energy efficiency of 1.2% were achieved thanks to the excitation of carriers by Cu LSP resonance toward the Zn sites, where water dissociation occurred with a lower *E*_a_ compared to a pure Cu surface.

A further example consists in the modification of Cu_2_O nanocubes, which are well-known photocatalysts for the partial oxidation of polypropylene [72], with plasmonic Au NPs (∼30 nm diameter) to selectively yield acrolein instead of propylene oxide and avoid the over-oxidation to CO_2_ (Figure 2d) [73]. Compared to pure Cu_2_O, the selectivity toward acrolein increased from ∼30 to ∼50% in the dark at 150 °C and further to ∼80% under visible-light illumination (Figure 2e). At the same time, the reaction rate and the conversion increased too (from 0.08 to 1.5%). This work further presented an interesting strategy to distinguish the effects of heating and hot electron transfer by coating the Au NPs with a 5 nm-thin SiO_2_ shell (near field effects may also come into play [74] but were not explicitly considered in that study). Experiments at increasing temperature with Au@SiO_2_–Cu_2_O catalyst showed an increase of both acrolein and propylene oxide formation compared to pure Cu_2_O cubes, thus suppressing the over-oxidation to CO_2_, but only the Au–Cu_2_O catalyst selectively yielded acrolein (Figure 2f). The suppression of hot electron transfer effects to univocally isolate purely thermal ones by a catalytically-inactive phase has been proven in other studies [56, 75] and is expected to play a relevant role in other material systems, such as in the case of metasurfaces.

## 3 Metasurfaces for light-to-heat conversion and hot carrier photogeneration

**Figure 4:**
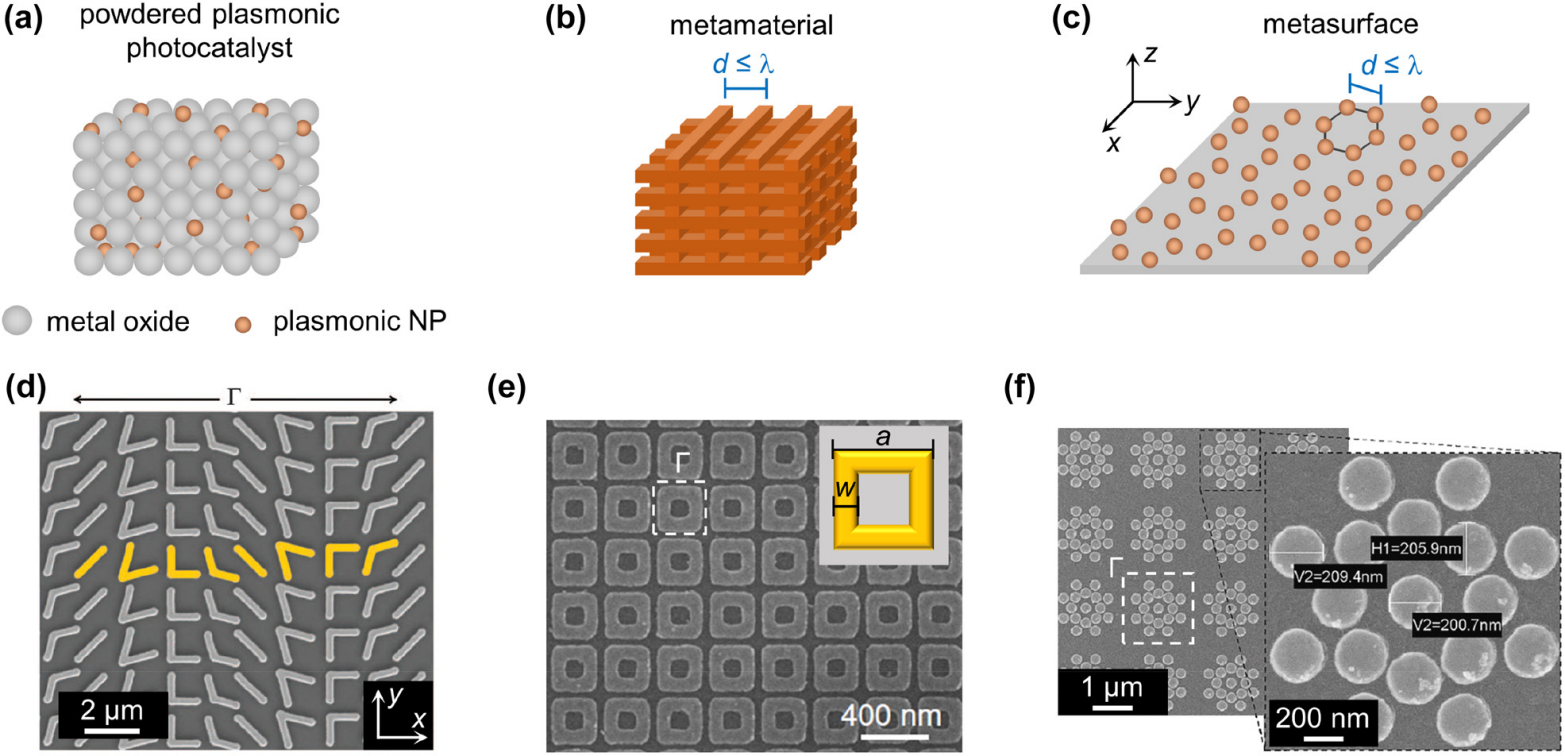
Schematic illustration of (a) a conventional powdered plasmonic photocatalyst, (b) a metamaterial, and (c) a metasurface made of plasmonic NPs arranged in a hexagonal lattice, which are all compared to the typical wavelength size (i.e., few hundreds of nm for solar-light applications). (d) Scanning electron microscope (SEM) image of a plasmonic metasurface made of Au V-shaped nano-units (∼220 nm width and ∼50 nm thickness) fabricated on a Si wafer. The unit cell repeats with a periodicity Γ = 11 µm in the *x* direction and 1.5 µm in the *y* direction. Adapted with permission from ref. [83]. Copyright 2011, American Association for the Advancement of Science. (e) SEM image of a TiN metasurface with Γ = 300 nm periodicity (square lattice). The square ring 30 nm-thick resonators (side *a* = 250 nm and width *w* = 50 nm, see inset) were grown on a SiO_2_/TiN stack with 60 nm and 150 nm thickness, respectively. Adapted with permission from ref. [84]. Copyright 2014, John Wiley and Sons. (f) SEM image of a metasurface made of Au flower-like nanoheaters with Γ = 1.5 µm periodicity (square lattice). The magnified view shows the details of the single flower-like unit made of Au nanodisks with diameter ∼200 nm. Adapted with permission from ref. [87]. Copyright 2021 author(s), licensed under a CC-BY Creative Commons Attribution 4.0 License.

As discussed in the previous section, the most typical powdered plasmonic systems consist of a mm-thick pellet made of plasmonic NPs randomly dispersed in a metal oxide matrix (Figure 4a). If neither the NPs nor the matrix exhibits an intrinsic catalytic activity, an additional metal coupled with the plasmonic nanostructures can provide the catalytic functionalization. This simple, anisotropic and disordered arrangement substantially differs from a metamaterial (Figure 4b), which can be defined as an artificial structure composed of subwavelength arrays of optical elements. The resulting ordered nanostructured material exhibits properties dictated by the behavior of the individual unit cell and an inhomogeneity scale that is much smaller than the wavelength of interest [76]. The schematic illustration in Figure 4b shows an example of three-dimensional (3D) metamaterial made of ordered wire-like structures having a sub-wavelength cross-section. Scaling such concepts from the 3D to the two-dimensional (2D) case has led to the development of metasurfaces, namely ordered quasi-2D arrays of subwavelength-spaced and optically thin nano-resonators (referred to as meta-atoms), with both characteristic size and periodicity of a few hundreds of nm (Figure 4c) [18, 19]. Note that, while the individual nano-resonators can be made of any material [77], here we focus on plasmonic metasurfaces, where the meta-atoms are metallic nanostructures (dielectric metasurfaces have been thoroughly discussed elsewhere [78]). The closeness of the meta-atoms leads to hybridization of the individual unit optical resonances (i.e., coupled dipoles in the simplest scenario of small NPs). In addition, the ordered arrangement of nanostructures can promote further resonant features dictated by the array periodicity. For example, surface lattice resonances or bound states in the continuum (BICs) can arise, introducing spectral features much sharper than the plasmonic resonances associated with the individual meta-atoms. Such effects may arise even in the case of nanostructures elongated along the out-of-plane (*z*) direction. Therefore, we include in the definition of metasurfaces also structures with a few hundreds of nm thickness, such as nanorods, nanopillars, or nanotubes [79, 80]. Moreover, metasurfaces can also feature periodicity in one direction only, namely consisting of 1D arrays (e.g., in the *x* direction) of ordered nanostructures with translational invariance in the other (e.g. *y*) direction, as for instance with nanowires [46, 81, 82].

The geometrical configuration of the individual meta-atoms and the onset of collective effects among nearest neighbors in the array are two critical unique features that enable to control electromagnetic radiation and implement advanced optical functionalities down to the nanoscale, making metasurfaces pivotal in various research fields. Among the numerous examples from literature, Figure 4d shows one of the seminal results: an array of Au V-shaped nano-resonators fabricated on a Si wafer with periodicity Γ = 11 µm in the *x* direction and 1.5 µm in the *y* direction [83]. Such metasurface was used to imprint a phase discontinuity on the impinging light, which then allowed light beam manipulation and generation of optical vortices, opening the way to the novel concept of metalenses and fostering a paradigm shift in nanophotonics toward flat optics [24]. Figure 4e, instead, shows an array of square-ring 30 nm-thick TiN resonators with 250 nm side and 300 nm periodicity grown on a SiO_2_/TiN stack (with 60 and 150 nm thickness, respectively) [84]. Such structure exhibited a broadband solar light absorption and high-temperature durability (up to 800 °C), both critical features for solar thermophotovoltaics. Figure 4f, finally, shows a metasurface made of Au flower-like nanoheaters, in turn made of 200 nm nanodisks assemblies, which behaved as a single plasmonic macro-unit thanks to the strong interaction between single units, therefore enhancing the photothermal effect of the metasurface. Such thermoplasmonic structures are of interest in various fields, such as solar energy harvesting (including photothermal catalysis), nano-medicine, water desalination, and three-dimensional printing [85, 86].

We now discuss the key features of plasmonic metasurfaces that can lead to their optimized exploitation in the field of photothermal catalysis and highlight the most relevant differences with powdered plasmonic systems.

### 3.1 Thermal effects

**Figure 5:**
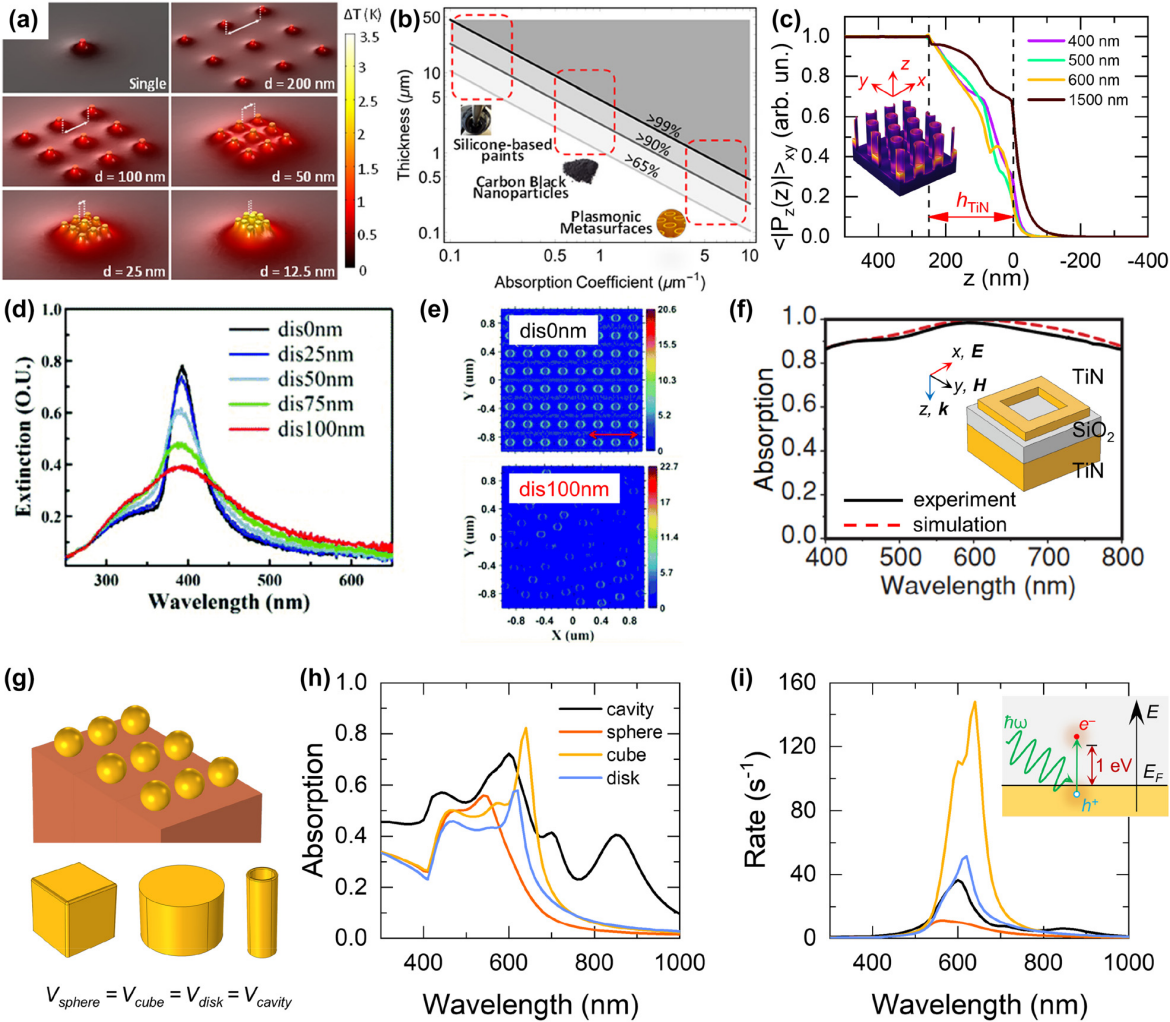
Geometric control of metasurfaces. (a) Calculated temperature profiles of a single and arrays of nine Ag NPs (7.5 nm radius) surrounded by water in a square lattice with lateral distances from 5 to 200 nm under light irradiation of ∼21.4 MW m^−2^. Adapted with permission from ref. [17]. Copyright 2020, American Chemical Society. (b) Thickness of a homogeneous layer of various materials, i.e., Pyromark 2500 [103] carbon black NPs [104], and plasmonic metasurfaces [30], required to absorb 65%, 90% and 99% of solar radiation (typical values of absorption coefficients were averaged over the solar spectrum). Adapted with permission from ref. [17]. Copyright 2020, American Chemical Society. (c) Modulus of the vertical component of the Poynting vector, |*P*_*z*_|, averaged over horizontal (*xy*) planes as a function of the thickness of simulated TiN nanocavities on Ti substrate (*z*-axis), normalized to its incident value for four different wavelengths. The inset shows the geometry of the array of TiN nanocavities colored according to the simulated power absorption upon plane wave illumination. Adapted with permission from ref. [27]. Copyright 2021, Elsevier. (d) Extinction spectra for Al nanodisk arrays (50 nm radius, 250 nm base pitch) with different degrees of displacement order, and (e) electric field maps under 378 nm excitation with *x* polarization for the perfectly ordered case (top) and maximum disordered (bottom). Adapted with permission from ref. [105]. Copyright 2020, Royal Society of Chemistry. (f) Simulated (dashed line) and measured (solid line) absorption spectra of a three-layer TiN/SiO_2_/TiN (30 nm/60 nm/150 nm thickness) metasurface. Corresponding to the SEM image in Figure 4e. Adapted with permission from ref. [84]. Copyright 2014, John Wiley and Sons. (g–i) Numerical simulation of hot electron generation rate in metasurfaces. (g) Schematic of the considered metasurface, consisting of a rectangular array of Au nanoparticles on Al_2_O_3_ substrate. Spheres (radius 60 nm), cubes, disks, and cavities are considered by keeping the unit cell’s metal volume constant. (h) Absorption spectra of the metasurfaces corresponding to the different geometries considered. (i) Hot electron generation rate computed according to ref. [118], [119], [120] for the metasurfaces under consideration for an exemplary excess energy of 1 eV above the Fermi level (the inset provides a schematical illustration).

Closely-packed nano-units display larger temperature increase under irradiation compared to the same farther apart, as shown when inspecting the spatial profile of temperature across an exemplary finite ordered ensemble of a few (nine) Ag NPs (Figure 5a). In general, upon standard illumination conditions, isolated NPs produce a relatively small temperature increase (typically, a few µK for ∼10^2^ W m^−2^), mainly localized around the NP surface and decaying as ∼1/*r*, where *r* is the radial coordinate outside of the nano-object [88, 89]. On the contrary, close-by NPs outperform the isolated case under the same irradiation conditions, as the individual heating contributions sum up. The resulting temperature distribution features thus higher temperatures due to collective effects (compare panels in Figure 5a). When a large number of NPs is employed, the precise spatial arrangement of the individual nano-heaters impacts the profile of the photoinduced temperature increase to a lesser degree, and the excited structure can be described as an effective homogeneous dissipater. This aspect has been thoroughly addressed in previous reports [90], [91], [92]. For powdered systems, this is the most common scenario, therefore the material thermal properties (e.g. conductivity) and the concentration of NPs are the only degree of freedom possibly promoting the light-induced heating. Conversely, the photothermal process can be engineered and enhanced in metasurfaces, thus achieving thermal dissipations (heat power densities) much higher than conventional plasmonic NPs randomly dispersed in an embedding matrix [93]. The meta-atom morphology and spatial orientation, the material supporting the meta-atoms, the array periodicity and interaction between neighbors, together with the metasurface overall dimension and optical response become relevant parameters for the design of optimal nanostructures. Besides, the control over the configurational characteristics of ordered systems may lead to thermal profiles which depend on the meta-atom spatial arrangement. Unlike nanocomposite powders and despite the relatively large number of individual heaters, metasurfaces can be tailored to produce pronounced thermal hot spots and gradients at the nanoscale [94]. Suitable NPs (i.e., bowties or nanocones, recently shown to support thermal gradients both in the stationary [95] and ultrafast [96, 97] regime of photoexcitation) can be chosen and appropriately oriented in space to generate inhomogeneous heating and material distributions to tailor their interaction with reactants. Note that, following the very high temperatures which can be generated under moderate light concentration regimes, NP local melting and reshaping can be induced [98, 99] unless refractory materials such as transition metal nitrides – TMNs – (i.e., TiN, ZrN, or HfN) [84, 100], refractory metals [101], or conventional metals coated with refractory oxide layers [102] are employed.

### 3.2 Enhanced absorption coefficient

The strong electromagnetic field confinement of plasmonic metasurfaces makes it possible to achieve complete light absorption within a sub-wavelength thickness [20], [21], [22]. Unlike mm-thick powdered pellets, nanopatterned materials enable indeed to induce dissipation power densities much closer to the illuminated surface, due to the high localization of the light–matter interaction. Note that, in these terms, the effect of electromagnetic dissipation confinement does not strictly require the meta-atoms to be ultrathin, but it rather results from the metasurface capability to produce a quasi-2D dissipation. This justifies the use of elongated meta-atoms (tubes, pillars, etc.), as long as radiation is efficiently absorbed in a few hundred nm. Evidently, randomly dispersed NPs do not offer the same potentiality as metasurfaces, which instead can maximize thermal and nonthermal gradients at the illuminated surface, possibly fostering heat and hot carrier transfer toward reactants. In this regard, Figure 5b reports the minimum thickness required for a homogeneous layer with varying average absorption coefficient (in m^−1^) to absorb 65, 90 and 99% of solar radiation. Plasmonic metasurfaces [30] with <1 µm thickness offer the same performance as black paints [103] or carbon black NPs [104] with orders of magnitude higher thicknesses. The same concept is further illustrated in Figure 5c, which shows how a metasurface composed of 250 nm-thick TiN nanocavities can efficiently absorb light (more details on such structure are reported below and in Section 4) [27]. In particular, the graph reports the simulated spatial profile of the Poynting vector vertical (*z*) component (normalized to its incident value and averaged over horizontal *x*–*y* planes) of a plane wave impinging at normal incidence on the structure, evaluated along the direction normal to the array. The individual cavity thickness (*h*_TiN_ = 250 nm) is highlighted. For wavelengths from the visible to the NIR range of the solar spectrum, the Poynting vector vertical component, which provides information on the electromagnetic energy flow associated with the incoming radiation, vanishes within the cavity thickness, demonstrating that the system absorbs light over a broadband spectrum in a few hundreds of nm.

Moreover, the high equivalent absorption coefficients offered by metasurfaces are expected to: (i) offer advantages in the time domain, to efficiently generate gradients at the illuminated surface. For instance, it has been recently shown [27] that, for a fixed dissipated power, limiting the dissipated thickness reduces the time required to reach the stationary conditions, accelerating the thermalization; and (ii) become particularly attractive when nonlinear (both optical and thermal) mechanisms are involved. Limited thicknesses featuring high absorption may enable photoinduced thermal and nonthermal effects *via* nonlinear phenomena upon much less extreme illumination regimes.

### 3.3 Broadband light absorption

The surface lattice resonance of simple plasmonic metasurfaces usually gives rise to a sharp absorption peak, as shown in Figure 5d in the case of an array of Al nanodisks with 50 nm radius. Narrow features are not ideal for efficient solar light harvesting. A possible strategy to broaden the optical absorption spectrum is introducing a controlled disorder in the metasurface [105], [106], [107]. This decreases amplitude and broadening of the absorption peak as the disorder degree increases, without significant shift from the resonance position, which remains close to ∼380 nm due to the unchanged average nanodisks period. The introduced disorder also breaks the symmetric distribution of the electric field excited at the resonant wavelength and leads to higher local maxima in correspondence of Al nanodisks very close to each other (Figure 5e). This concept can be further exploited to synthesize intrinsically disordered metasurfaces with high optical absorption, such as anodic alumina oxide (AAO) coated by Au [108] or TiN [109]. A different strategy toward broadband optical absorption consists in applying the approach of metal/insulator/metal (MIM) structures to the case of metasurfaces [21, 84, 110], [111], [112], as shown in Figure 5f for the case of TiN square-ring resonators grown on a SiO_2_ interlayer and a compact TiN back-reflector (see the corresponding SEM image in Figure 4e) [84]. This effect is further enhanced if the topmost metasurface layer is made of a dissipative metallic material (such as Ti, TiN, Ni, etc.) instead of noble metals [110, 112, 113]. The approaches mentioned above can also be combined to realize ultrathin absorbers made of a disordered metasurface layer, an oxide interlayer and a flat metallic back-reflector [114], [115], [116], [117]. Such a fine engineering of the absorption spectrum is not possible in the case of powdered plasmonic systems, in which the optical absorption is substantially dictated by the mass fraction between the plasmonic NPs and the oxide matrix.

### 3.4 Hot-carrier generation

Contrarily to the case of thermal effects, the choice of a specific geometry for the individual meta-atom in a metasurface is expected to have a major role in local phenomena occurring at the metal interface, such as the generation of non-thermal carriers. To illustrate this effect, we have investigated some exemplary metasurfaces based on Au nanoparticles lying on a Al_2_O_3_ substrate by varying the geometry of the individual meta-atom (Figure 5g). The comparison is performed between common nanoparticle geometries such as spheres, cubes, disks, and cavities, keeping the metal volume within the unit cell constant. The four structures in the metasurface exhibit substantially comparable absorption spectra, regardless of the features arising from the specific plasmonic modes supported by the different geometries (Figure 5h). Consequently, the absorption of radiation produces a thermal spatial profile that, for the structures, is essentially equivalent at the macroscale. Conversely, the investigated metasurfaces show significantly different hot carrier generation rate behaviors. To assess the generation rate of electrons potentially relevant for photocatalytic applications, we have implemented calculations proposed in refs. [118], [119], [120] for an excess energy of 1 eV above the Fermi level (i.e., an exemplary energy to populate anti-bonding orbitals of adsorbed reactive molecules) and an incoming intensity corresponding to a unitary linearly-polarized electric field (Figure 5i). Our numerical results clearly display the benefit of working with cubes or, more generally, with geometries supporting electromagnetic hot spots thanks to the enhanced electric field at the vertices [118, 121], which promote the generation of high-energy electrons at the nano-object interface with the surrounding environment, including adsorbed reactive gas molecules [122]. Importantly, for an optimized geometry of the individual NP, the ordered arrangement of nanostructures in metasurfaces enables to further engineer and tailor the interaction with the NP surface and hot spots. This represents a promising advantage to systematically control hot carrier-mediated effects (e.g., recently reported for ultrafast all-optical modulation of light [123]) if compared to nanocomposite powders, where NPs are instead randomly dispersed. Moreover, the rational positioning of NPs in array configuration can be exploited to promote higher local field enhancements at the meta-atom surface with respect to the isolated case. This could optimize the hybridization of the single NP resonances in metasurfaces, thus increasing the rate of hot carrier generation.

**Figure 6:**
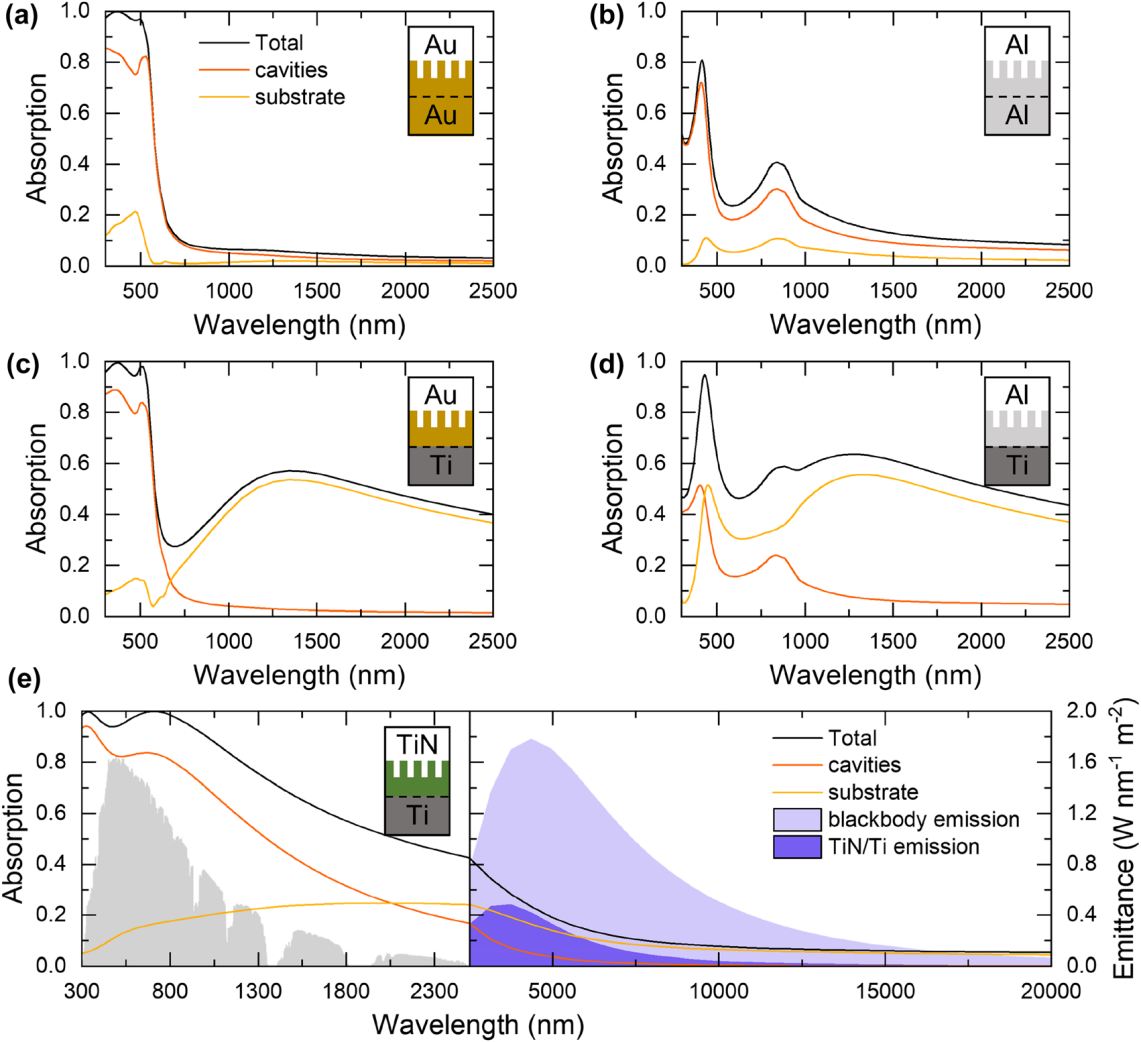
Optimization of light absorption in metasurfaces by materials choice. Absorption spectra of a 2D hexagonal array of cylindrical metasurfaces (external height = 250 nm, external radius = 45 nm, cavity depth = 162 nm, internal radius = 35 nm, and lattice constant = 165 nm) on a flat substrate; the disentangled contributions for the two layers are also reported. Different representative material combinations are reported: (a) Au/Au, (b) Al/Al, (c) Au/Ti, (d) Al/Ti, and (e) TiN/Ti. Panel (e) also reports the spectral solar irradiance (ASTM 1.5G) and the emittance of the TiN/Ti structure compared to a perfect blackbody material, both evaluated at 400 °C (a typical operative temperature in gas-phase photocatalysis conditions) by Planck’s formula. Permittivity data for Au and Al taken from [130], for Ti taken from [126], and for TiN taken from [131].

Based on the above considerations, we further discuss the flexibility of optical properties and, more specifically, the optimization of the absorption spectrum of plasmonic metasurfaces from narrow-band to broadband in view of sun-driven applications, by combining materials with different permittivity. As a case study, we performed numerical simulations for an exemplary geometry consisting of a hexagonal 2D array of nanocavities with 250 nm thickness on a flat substrate (Figure 6, see also Figure 5c), consistent with previous studies [27, 30] (see also Section 4). In particular, the total absorption spectra of two-layered nanocavities/substrate metasurfaces are reported together with the disentanglement of the individual contributions of the nanocavities and the substrate. For example, a 2D array of Au nanocavities lying on an Au substrate exhibits nearly-unitary absorption below 500 nm (Figure 6a), while for an Al/Al structure two main peaks are found at ∼400 and ∼800 nm (Figure 6b). These features can be ascribed to a combination of different absorption phenomena. On the one hand, cavity modes can be excited at UV/visible wavelengths; on the other hand, interband transitions occur in the UV for Au [124] and around 800 nm for Al [125]. Therefore, in the case of Au/Au structure, these contributions superimpose to each other (Figure 6a), while in the case of Al/Al structure they give rise to two well-defined absorption peaks (Figure 6b). For the latter case, in addition, the optical absorption exceeds 20% on a much broader spectral range compared to the Au/Au case (up to ∼1250 nm). Interestingly, this effect can be optimized by choosing a different substrate, thus further extending the range of light absorption. For example, by employing Ti to replace either Au or Al underlying the cavities (Figure 6c and d, respectively), a broad absorption peak in the 1000–1500 nm region arises because of a wider range for interband absorption in transition metals like Ti [126]. As a result, both the Au/Ti and Al/Ti structures absorb more than 40% from ∼1 to ∼2.5 µm owing to dissipation occurring in the substrate. Finally, by choosing TiN as the material for the nanocavity layer on top of a Ti substrate, the absorption spectrum gets further broadened and improved, with values >90% in the whole UV–visible range and >40% up to 2.5 µm (Figure 6e). This effect can be attributed to the broader energy range of interband absorption in TiN compared to Au [127]. The TiN/Ti combination offers thus an optimal absorption spectrum compared to the solar irradiance (also reported in Figure 6e). Furthermore, the absorption monotonically decreases in the IR range and the contribution of the nanocavity layer becomes negligible for *λ* > 10 µm (Figure 6e, right panel). The medium-IR (MIR) spectral region is still interesting because it is the typical thermal radiation range for a surface heated at a few hundred °C. Kirchhoff’s law of thermal radiation states the equality between spectral emissivity and optical absorption, so that a low absorption in the MIR range allows limiting the radiative losses. This concept is a fundamental feature of the so-called spectrally selective absorbers employed in solar thermophotovoltaics [8, 128]. Interestingly, the TiN nanocavities/Ti substrate combination features a high absorption in the UV–visible–NIR range as well as a low absorption in the MIR range, leading to a low emittance at 400 °C (as a representative temperature for gas-phase photocatalysis experiments) compared to a perfect blackbody at the same temperature (Figure 6e, right panel). Therefore, this feature could be critical to reach high temperatures for gas-phase catalytic reactions under moderate solar concentration factors [21]. Overall, Figure 6 highlights the importance of a proper material choice in the design of metasurfaces with optimal photothermal properties, a non-trivial issue requiring careful calculations even in the simpler geometry of two flat metal layers, as recently discussed [129].

## 4 Photothermal catalysis with plasmonic metasurfaces

**Figure 7:**
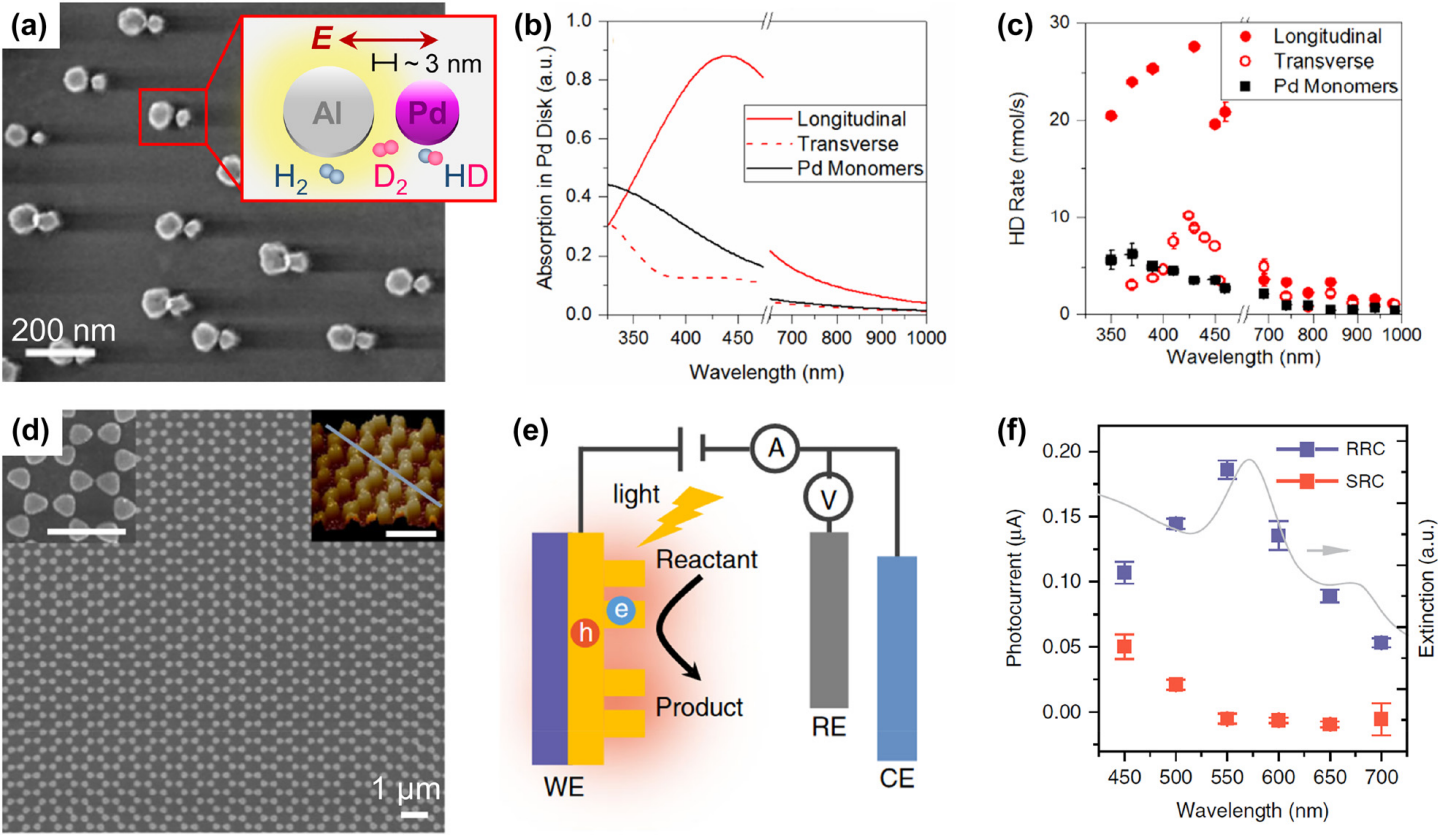
First implementations of plasmonic metasurfaces in catalysis. (a–c) Al–Pd antenna–reactor heterodimers with 3 nm gaps for the photocatalytic hydrogen dissociation reaction: (a) top-view SEM image and schematic, (b) optical absorption in the Pd units, and (c) and wavelength-dependent absorption HD production rate under longitudinal and transverse excitation, compared with the same obtained with Pd monomers. Adapted with permission from ref. [28]. Copyright 2016, American Chemical Society. (d–e) Plasmonic Au bowtie array photoelectrode: (d) SEM and atomic force microscopy (AFM) images (scale bars: 1 µm), (e) schematic of the three-electrode electrochemical system (WE: working electrode; RE: reference electrode; CE: counter electrode), and (f) rapid response current (RRC) and slow response current (SRC) as a function of wavelength compared to the extinction spectrum of the array. Adapted with permission from ref. [132]. Copyright 2019 author(s), licensed under a CC-BY Creative Commons attribution 4.0 License.

Early reports shifting paradigm in plasmonic photothermal catalysis from isolated plasmonic NPs toward plasmonic metasurfaces report the fabrication of antenna–reactor nanodisks on flat substrates by hole-mask colloidal lithography [28, 29]. For example, Al–Pd antenna–reactor heterodimers were studied for the photocatalytic hydrogen dissociation reaction, i.e., H_2_ + D_2_ → 2HD, where D is deuterium (Figure 7a) [28]. Al and Pd nanodisks with ∼35 nm heights and diameters of ∼75 and ∼50 nm, respectively, were separated by either “small” or “big” gaps, i.e., ∼3 and ∼8.6 nm. Under laser light illumination with longitudinal polarization (i.e., with the polarization parallel to the inter-particle axis), the short gaps facilitated the excitation of the otherwise weak dipolar plasmon in Pd (the reactor unit) by the electric field of the Al dipolar plasmon (antenna unit, inset of Figure 7a), leading to a broad absorption peak at ∼460 nm (Figure 7b). The same did not occur under transverse polarization (i.e., with the polarization perpendicular to the inter-particle axis). The interaction between the antenna and reactor units under longitudinal polarization promoted a higher H_2_ dissociation rate compared to that under transverse polarization, with a trend of the HD production rate versus wavelength closely resembling the absorption spectra (Figure 7c). The maximum reaction rate of ∼3.6·10^7^ mmol g_cat_^−1^ h^−1^ under 200 W cm^−2^ intensity (430 nm) was found. In the case of metasurfaces, the reaction rate may be normalized by the illuminated (total geometrical) areas instead of by the catalyst mass as in the case of photovoltaics, which in this case leads to the values of ∼346 (∼1.73) mol m^−2^ h^−1^ for the illuminated (total geometrical) areas of the sample.

A more recent study following a similar experimental strategy further showed that Au@SiO_2_–Pt antenna–reactor arrays could be used for the CO oxidation reaction [29], CO + 1/2O_2_ → CO_2_, which is frequently used as a model reaction and is important for the removal of CO from fuel cells. A ∼10 nm-thick SiO_2_ interlayer was added to prevent alloy or intermetallic phase formation during thermal treatments, which were performed at 350 °C in the same experimental conditions for the CO oxidation reaction, thus showing how a thin oxide interlayer can stabilize metasurfaces under realistic photothermal catalysis conditions.

Guidance on future gas-phase experiments with plasmonic metasurfaces may be provided by the closely-related field of photoelectrochemistry, which allows assessing the capability of a photoelectrode to drive oxidative or reductive reactions under the application of an external bias. Photoelectrodes based on plasmonic metasurfaces have indeed been recently reported [132], [133], [134]. For example, a plasmonic photoelectrode based on Au bowtie array with 600 nm periodicity (Figure 7d) was tested as working electrode under visible-light illumination (*λ* > 420 nm) in a three-electrode cell at neutral pH (Figure 7e) [132]. First, such a plasmonic metasurface photoelectrode could work either as photoanode or photocathode, which is not the case for conventional semiconductor-based photoelectrodes, demonstrating the high versatility of plasmonic metasurfaces toward various catalytic reactions. Furthermore, chronoamperometry experiments discerned two components of the overall photocurrent, i.e., a rapid response current (RRC) and a slow response current (SRC). The former exhibited a wavelength dependence resembling the extinction spectrum of the plasmonic photoelectrode, on the contrary of the latter (Figure 7f). As a consequence, the RRC was attributed to the hot electron transfer process, while the SRC to purely thermal effects, thus illustrating a strategy to decouple such effects regardless the specific catalytic reaction of interest.

**Figure 8:**
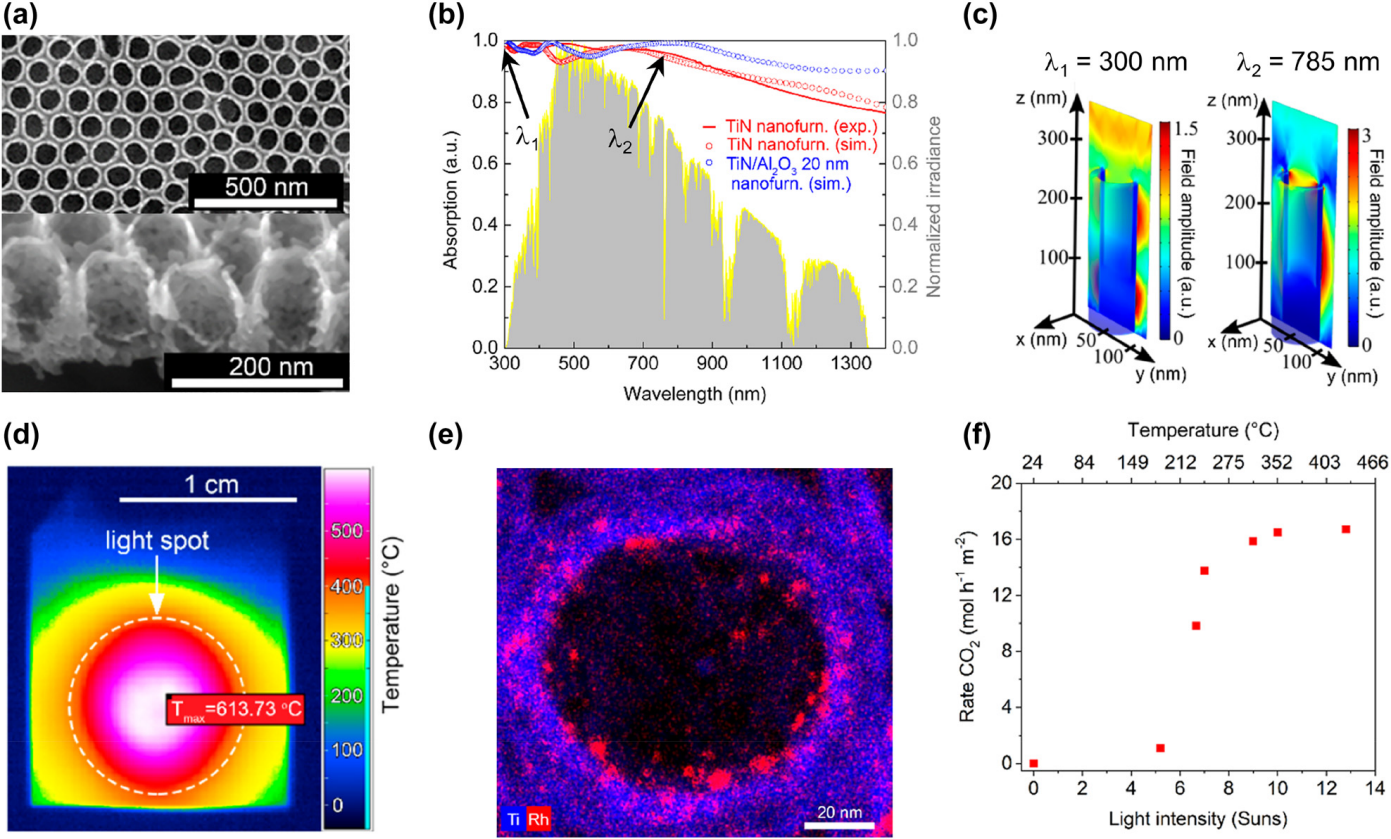
TiN nanofurnaces as broadband and ultrathin solar absorbers for photothermal catalysis. (a) Top-view (top) and cross-sectional (bottom) SEM images. (b) Absorption spectra (experimental: solid red line; simulated: hollow red circles; simulated with 20 nm Al_2_O_3_ overlayer: hollow blue circles) compared to the normalized irradiance of the AM 1.5G solar spectrum. (c) Simulated electric field amplitude distribution under *λ*_1_ = 300 nm and *λ*_2_ = 785 nm excitation wavelengths (marked in b). (d) Infrared (IR) thermal camera image of a TiN nanofurnace under 19 Suns illumination. (e) Elemental map of a single TiN nanofurnace decorated with Rh nanoparticles. (f) Rate of CO_2_ production from CO oxidation at different solar light intensities and corresponding generated temperatures measured using the IR thermal camera. Adapted with permission from ref. [30]. Copyright 2020, American Chemical Society.

A recent work has shown a potential step forward the realistic use of plasmonic metasurfaces for photothermal catalysis by a 2D hexagonal array of TiN cylindrical nanocavities tested for the CO oxidation reaction (Figure 8) [30]. This work offered a number of insights both from a materials science and reactor engineering points of view. A long-range self-ordering with a hexagonal lattice (nanocavity center-to-center distance of ∼100 nm) could be obtained by anodizing Ti plates, without the need for surface patterning [135], followed by nitridation in NH_3_ at 600 °C (Figure 8a). Such metasurface exhibited broadband light absorption in the UV–visible–NIR range (Figure 8b), which can be explained as a combination of multiple resonances coupled with the lossy nature of TiN (see also the discussion of Section 3 and, in particular, Figure 6e). The resonant phenomena excited by this geometry consisted in pure cavity modes (*λ*_1_ = 300 nm), hybrid modes (first-ordered cavity mode and SPP waves propagating along the cavity walls, *λ*_2_ = 785 nm), both illustrated in Figure 8c, and LSPR at the cavity corners (*λ*_3_ = 1500 nm). The broadband absorption together with the refractory nature of TiN allowed the nanocavities to reach the remarkable temperature of ∼610 °C under 19 Suns illumination (Figure 8d). This led to oxidation in environmental conditions, which was instead limited upon catalysis experiments in the reactive gas mixture. Nevertheless, surface oxidation of TiN is not expected in the case of reactions involving reducing processes and may be circumvented by depositing an Al_2_O_3_ overlayer, which, according to numerical simulations, could further improve the optical absorption (Figure 8b). The temperature generated under irradiation could be measured by an infrared (IR) thermal camera viewing the sample back surface thanks to the use of a thin (125 µm) Ti substrate (Figure 8d), which could avoid the issue of placing multiple thermocouples along the sample thickness to track thermal gradients along the *z* direction, as typical of powdered plasmonic systems (as discussed in Section 2.2). TiN nanocavities could not drive alone the CO oxidation reaction, therefore they were decorated by Rh NPs (3–5 nm diameter, Figure 8e) and produced the highest amount of CO_2_ of ∼16 mol m^−2^ h^−1^ (normalization by the total geometrical area of the sample) for intensities higher than ∼9 Suns (Figure 8f), with a sigmoidal behavior of the reaction rate typical of thermally-activated processes. The solar-to-heat conversion efficiency was evaluated as

(3) $$\eta_{\text{STP}}=\left(P_{\text{in}}-P_{\text{conv}}-P_{\text{rad}}\right)/P_{\text{in}}$$

where *P*_in_, *P*_conv_, and *P*_rad_ are the power of the incident light, the power lost due to convection and the power lost due to radiation, respectively. The values of *η*_STP_ ∼35% in air and ∼68% in vacuum (*P*_conv_ = 0) were found assuming negligible conductive losses.

Compared to the metasurfaces presented in Figure 7, TiN nanocavities (Figure 8) offered a higher surface area thanks to the porous tube morphology and could develop high temperatures and high reaction rates (with Rh catalyst) under much lower light intensity (i.e., ∼2 vs. 200 W cm^−2^). Therefore, the latter seem to represent a suitable platform for follow-up studies that may focus on more interesting reactions from the energy conversion point of view, such as the CO_2_ reduction reaction or Fischer–Tropsch processes.

## 5 Outlook

Plasmonic metasurfaces have been, so far, mostly neglected in the field of photothermal catalysis as opposed to powdered plasmonic systems, which closely resemble the catalysts employed in industry. Only a handful of papers involving the application of plasmonic metasurfaces toward catalytic reactions have been reported. Therefore, a wide room for improvement is available for such nanomaterials in this field, both from the experimental and theoretical point of view.

First, we note that “traditional” fabrication processes for metasurfaces, such as electron beam lithography (EBL) and focused ion beam (FIB) writing [136], are affected by low throughput and high costs compared to the cheap processes required by powdered plasmonic systems. However, substantial progress has been made toward the large-scale fabrication of metasurfaces [137, 138]. For example, plasmonic metasurfaces have been realized by photolithography [138], a mature technology in complementary metal-oxide semiconductor (CMOS) manufacturing, such as 50 nm-thick Au resonators on SiO_2_ for metalenses [139], and by nanoimprint lithography (NIL) [137], such as Al disks on SiO_2_ as IR filters [140], Au strips on polymeric substrates as plasmonic sensors [141] and, remarkably, hot-carrier-driven Au/TiO_2_ photoelectrodes [142]. Such methods are also compatible with flexible substrates, which currently cannot withstand the relatively high temperatures of gas-phase catalytic processes. However, polytetrafluoroethylene (PTFE) might be explored as a substrate thanks to its low thermal conductivity and chemical inertness under low light concentration regimes. On the other hand, partially disordered metasurfaces can be obtained by even cheaper fabrication processes and feature a nearly-perfect optical absorption [109, 116, 117]. On the other hand, ordered metasurfaces made of meta-atoms with sharp corners (such as nanocubes or nanostars) should be employed if strong electronic or electromagnetic field effects are sought [118, 121].

Related to the above, large-scale development necessarily comes along with a thoughtful material selection, as encouraged by official guidelines, such as the Critical Raw Materials Resilience by the European Commission [143]. TMNs have steadily emerged as alternative plasmonic materials, thanks to their compatibility with CMOS fabrication process and higher abundance, thermo-mechanical stability, and optical tunability compared to noble metals [100, 144]. The thermal stability of TiN metasurfaces has been for instance clearly assessed [84]. Notably, TiN nanocavities could represent a viable choice for further studies thanks to the scalable synthetic route (i.e., anodization followed by thermal treatments) without compromising a high degree of long-range order [30]. It is anticipated that other TMNs, such as ZrN, HfN, or ternary nitrides, might also be prepared in a similar fashion. Alternatively, Al_2_O_3_ nanotube arrays, which can also be prepared by anodization, might be used as substrates for further deposition of plasmonic overlayers [108, 109, 145]. However, these materials are not catalytically active, thus requiring functionalization through, for example, the addition of metals, such as Pt, Pd, Rh, Ru, etc. Notably, lowering the content of metals is a high priority in modern catalyst design with an exponential increase of successful stories of ultra-small metal NPs and, even better, single atom-based catalysts [146, 147]. Due to the limited thickness required by metasurfaces to achieve near-unitary absorption and by the small volume required by the catalytic units, if present, the constraints on material selection can be therefore mitigated.

An additional motivation for pursuing further studies on TMN-based plasmonic metasurfaces for photothermal catalysis is the possibility of supporting transient thermal hot-spots [96, 97]. Ultrafast reflectance experiments showed that TiN and ZrN have a substantially higher electron-phonon coupling coefficient compared to Au, i.e., *G* ∼1.0 × 10^18^ versus ∼2.8 × 10^16^ W m^−3^ K^−1^, respectively [148, 149]. Since TMNs also feature a lower thermal conductivity than that of Au (i.e., *κ* ∼29 vs. ∼300 W m^−1^ K^−1^ for TiN and Au at room temperature, respectively) [150], under ps-pulsed laser illumination a thermal hot spot can be formed in correspondence of an electromagnetic hot-spot, while the same does not occur in Au [96]. Due to the exponential dependence of the reaction rate on temperature by the Arrhenius law, thermal hot spots in TMNs can produce ∼10^3^ more product than Au despite their lower intrinsic catalytic activity [96]. These recent findings may open the way to photothermal catalysis experiments irradiating plasmonic metasurfaces with ultrashort pulses and nanoscale resolution. Chiral plasmonic metasurfaces may be particularly suitable for such experiments, thanks to their local control on hot electron and thermal effects in different parts of the meta-atom, which depend on the light polarization [151, 152].

Critical studies investigating the disentanglement of thermal and non-thermal effects in photothermal catalysis based on powdered plasmonic systems have recently enlivened the community and affirmed the non-trivial influence of such effects in enhancing the reaction rate and selectivity in various chemical processes [57], [58], [59], [60], [61], [62], [63], [64], [65], [66]. Following the numerical approaches presented in Figure 5, plasmonic metasurfaces may be thoroughly investigated from the theoretical point of view to assess the relative importance of thermal and non-thermal effects for a specific combination of chemical reaction and metasurface geometry. As mentioned above, meta-atoms featuring sharp edges should specifically employed to generate larger hot electron densities than “smoother” counterparts, i.e., spheres or cavities [118, 121]. In all cases, collective thermal effects tend to promote substantially homogeneous temperatures [90] (apart from the specific cases of thermal hot spots discussed above). In this regard, plasmonic metasurfaces could offer an advantage compared to powdered plasmonic systems because 2D ordered models are more realistic representations of the experimental case, thus possibly facilitating the simulation of experimental results. Moreover, the quasi-2D nature of plasmonic metasurfaces may substantially limit thermal gradients along the sample thickness, which have led to significant issues in temperature measurements in the case of powdered plasmonic systems, while such gradients persist in the *xy* plane due to light focusing. Apart from numerical simulations of thermal gradients, the temperature should be experimentally measured by IR imaging techniques (i.e., by thermal cameras) that provide high-resolution thermal images of the sample surface. However, such techniques are typically more expensive than conventional thermocouples and require an accurate measurement of the surface emissivity, which is typically retrieved by reflectance measurements in the IR range by FTIR spectroscopy at room temperature. This intrinsically introduces an inaccuracy because of the dependence on temperature, which cannot be accounted for unless high-temperature spectral emissometers are employed [153].

The above point highlights the importance of assessing the optical properties of the catalyst material in the IR region of the electromagnetic spectrum because, by minimizing the emissivity in such region, higher temperatures and thus, optimal solar-to-heat conversion may be achieved. This point has been mostly investigated for solar-thermal applications [8, 128] but somehow overlooked so far in the field of photothermal catalysis. We therefore expect dedicated studies that may combine numerical (as in Figure 6) and experimental investigations to identify material combinations and geometrical arrangements to realize plasmonic metasurfaces with spectrally-selective absorption properties, i.e., near-unity absorption from the UV to the NIR regions and low absorption in the MIR region of the electromagnetic spectrum.

A further point that should be considered to allow large-scale development of plasmonic metasurfaces for photothermal catalysis reactors consists in a reliable benchmarking. In this regard, several quantities should be reported, starting from the reaction rate, which may be normalized by: (a) the illuminated area [28], which likely drives almost entirely the reaction in case of significant thermal gradients along the *xy* plane due to a focused light spot; (b) the total geometrical area of the sample [30], which is more significant to evaluate the overall amount of material and reactor area involved; or (c) the catalyst mass, which allows a closer comparison to powdered plasmonic systems. The selectivity (see Section 2.2) is also an important quantity to be assessed and it should be as close as possible to 100% for the reaction of interest. A formulation for the apparent quantum efficiency for non-thermal processes has also been introduced as [75]:

(4) $${\text{AQE}}_{\text{nt}}\left(\text{\%}\right)=\left[r_{\text{nt}}\left(\text{molecules }{\text{s}}^{-1}\right)/I\left(\text{photons }{\text{s}}^{-1}\right)\right]\times 100$$

where *r*_nt_ = *r*_tot_ − *r*_therm_ is the non-thermal reaction rate evaluated by subtracting the value measured in a dark control experiment to the total photothermal reaction rate, and *I* is the photon flux. As discussed in Section 2.2, such control experiment requires special attention to replicate the photothermal mechanism. Other works have evaluated the energy efficiency for the catalytic reaction [70, 71] (Section 2.2) or the solar-to-heat conversion efficiency [30] (Section 4). To combine photochemical and thermal efficiencies, a temperature-dependent dimensionless photothermal figure of merit has been proposed, and expressed as [12]:

(5) $$\text{PTF}\left(T\right)=\left[\eta_{\text{ct}}\left(T\text{*}\right)\text{·}\alpha \text{·}\kappa_{\text{int}}\left(T\text{*}\right)\text{·}T\text{*}\right]/\left[\kappa_{\text{cat}}\left(T\text{*}\right)\text{·}T\right]$$

where *α* is the fractional solar absorptance, *η*_ct_(T*) is the photochemical charge transfer efficiency at the non-equilibrium local temperature T*, *κ*_int_(T*) is the thermal conductivity of the interface between the nano-heater and the catalyst, *κ*_cat_(T*) is the thermal conductivity of the catalyst substrate, and *T* is the measured temperature of the catalyst bed. However, some parameters in the equation may not be easily retrieved by experiments. Moreover, gas-phase photothermal catalysis involves a variety of chemical reactions, including both spontaneous and non-spontaneous processes, making it harder to find a univocal rigorous metric. As an example, a tentative benchmarking was proposed in a recent work including three major fields of photothermal catalysis, i.e., solar fuel production, chemical synthesis and environmental remediation, which also involved chemical reactions in the liquid phase [154]. Even for the relatively well-developed field of photocatalytic water splitting efficiency testing protocols have not been found yet [155]. This is very different from the standardized and well-known efficiency graph for photovoltaic technologies published by National Renewable Energy Laboratory (NREL) [156]. Therefore, substantial progress needs to be made in the field of gas-phase photothermal catalysis, both involving powdered systems and metasurfaces (regardless the plasmonic nature), to develop rational benchmarking protocols that may be valid for a wide spectrum of chemical processes.

Finally, compared to powdered plasmonic systems, plasmonic metasurfaces present a lower active surface available for chemical reactions with the gas phase. This issue requires a different reactor design compared to the former case. For example, flat panel-like reactors may be developed, contrary to cylindrical reactors reminiscent of the vessels for dark thermal catalysis. In this regard, it is critical that metasurface-based materials generate temperatures as high as ∼500 °C under moderate concentration factors (i.e., ∼10 Suns) to avoid optics that would dramatically increase the reactor cost. For example, arrays of unexpensive optics (i.e., Fresnel lenses) may be employed. Such reactor design allows achieving strong focalization conditions leading to both optical and thermal nonlinearities, which can be beneficial for chemical reactions thanks to the exponential dependence on the reaction rate by Arrhenius formula. An example of a small-scaled reactor effectively exploiting thermal nonlinearities in a solar-driven process (water desalination) has been, for instance, recently reported [104]. In light of these considerations, a scalable synthetic process to prepare metasurfaces on large areas with Earth-abundant materials is even more important. All these aspects highlight several potential routes to investigate for enabling the deployment of metasurfaces for photothermal catalysis in the near future.
